# Noncanonical calcium binding motif controls folding of HopQ1, a *Pseudomonas syringae* type III secretion effector, in a pH-dependent manner

**DOI:** 10.1038/s41598-024-82848-z

**Published:** 2024-12-30

**Authors:** Fabian Giska, Wojciech Rymaszewski, Malgorzata Lichocka, Marcin Piechocki, Jakub Kwiatkowski, Jarosław Poznański, Magdalena Górecka, Magdalena Krzymowska

**Affiliations:** Institute of Biochemistry and Biophysics, Polish Academy of Sciences, Warsaw, Poland

**Keywords:** Pathogens, Plant immunity

## Abstract

Virulence of many gram-negative bacteria relies upon delivery of type three effectors into host cells. To pass through the conduit of secretion machinery the effectors need to acquire an extended conformation, and in many bacterial species specific chaperones assist in this process. In plant pathogenic bacterium *Pseudomonas syringae*, secretion of only few effectors requires the function of chaperones. This raises a question how chaperone-independent effectors achieve an appropriate conformation for the secretion. One such mechanism was previously described for AvrPto. It contains a pH-sensitive switch, which is involved in unfolding of the effector at the mildly acidic pH corresponding to the pH value of the bacterial cytosol, and refolding at the neutral pH. Therefore, it was proposed that the switch facilitates first translocation of AvrPto and then its maturation once the effector reaches the cytoplasm of host cell. Here we show that an atypical motif of HopQ1, another effector of *P. syringae*, reversibly binds calcium in pH-dependent manner, regulating the effector thermal stability. Therefore, we propose a model that HopQ1 traversing through the type three secretion system encounters conditions that maintain its extended conformation, while upon delivery into host cell the effector undergoes refolding.

## Introduction

Phytopathogenic bacteria synthesize a set of specific effector proteins that modify host physiology. The effectors act either within host cells or in the intercellular space (apoplast), and gram-negative bacteria mostly use type III secretion system (TTSS) to deliver them to their destination. TTSS consists of a basal body anchored in a bacterial cell envelope and a needle-like structure that together form a conduit for the effectors. Its lumen is very narrow with passages not exceeding few nm^1,2^, and therefore the effectors need to assume an extended conformation to be translocated. In many bacterial species specific chaperones operate at the entrance to this nanomachinery facilitating (partial) unfolding of their client effectors. In contrast, in *Pseudomonas syringae*, an economically important plant pathogenic bacterium, the repertoire of the TTSS chaperones is very scarce. Some chaperones can assist in the translocation of more than one effector while the most of the TTSS substrates are chaperone independent^3^. This implies that effectors in this species might have evolved other mechanisms to acquire a competent state for the secretion.

In this study, we analyzed properties of HopQ1 from *P. syringae* which is a chaperone independent effector^4^. HopQ1 and HopQ1-like proteins (HLPs) display homology to Nucleoside Hydrolases but they group as a distinct clan within this superfamily, which is distant from the classical core^5^. They all contain a modified calcium binding motif (DXXXDXDD) within the predicted catalytic center, compared to the canonical enzymes (DXDXXXDD). This suggests that HLPs may have evolved to process other substrates. Consistently, XopQ, a HopQ1 homolog from *Xanthomonas euvesicatoria* exerts 2′,3′-cAMP/cGMP phosphodiesterase activity, which is possibly employed by bacteria to hydrolyze signaling molecules produced by a subclass of plant resistance receptors^6^. Here we show that calcium plays an important role in the stabilization of HopQ1 structure. The affinity of the HopQ1 atypical binding motif for calcium changes in a pH-dependent manner, and therefore enables a pH-sensitive control of the effector thermal stability. A similar mechanism has been described for AvrPto from *P. syringae*. Protonation of the conserved histidine residue (H87) within AvrPto, under mildly acidic conditions corresponding to the pH of the bacterial cytoplasm mediates unfolding of the effector thus making it competent for the transport via TTSS^7^. We propose that in the case of HopQ1, the effector enters TTSS in the calcium-free state and adopts the mature conformation upon calcium binding in the host cell.

## Results

### HopQ1 forms oligomers

**Fig. 1:**
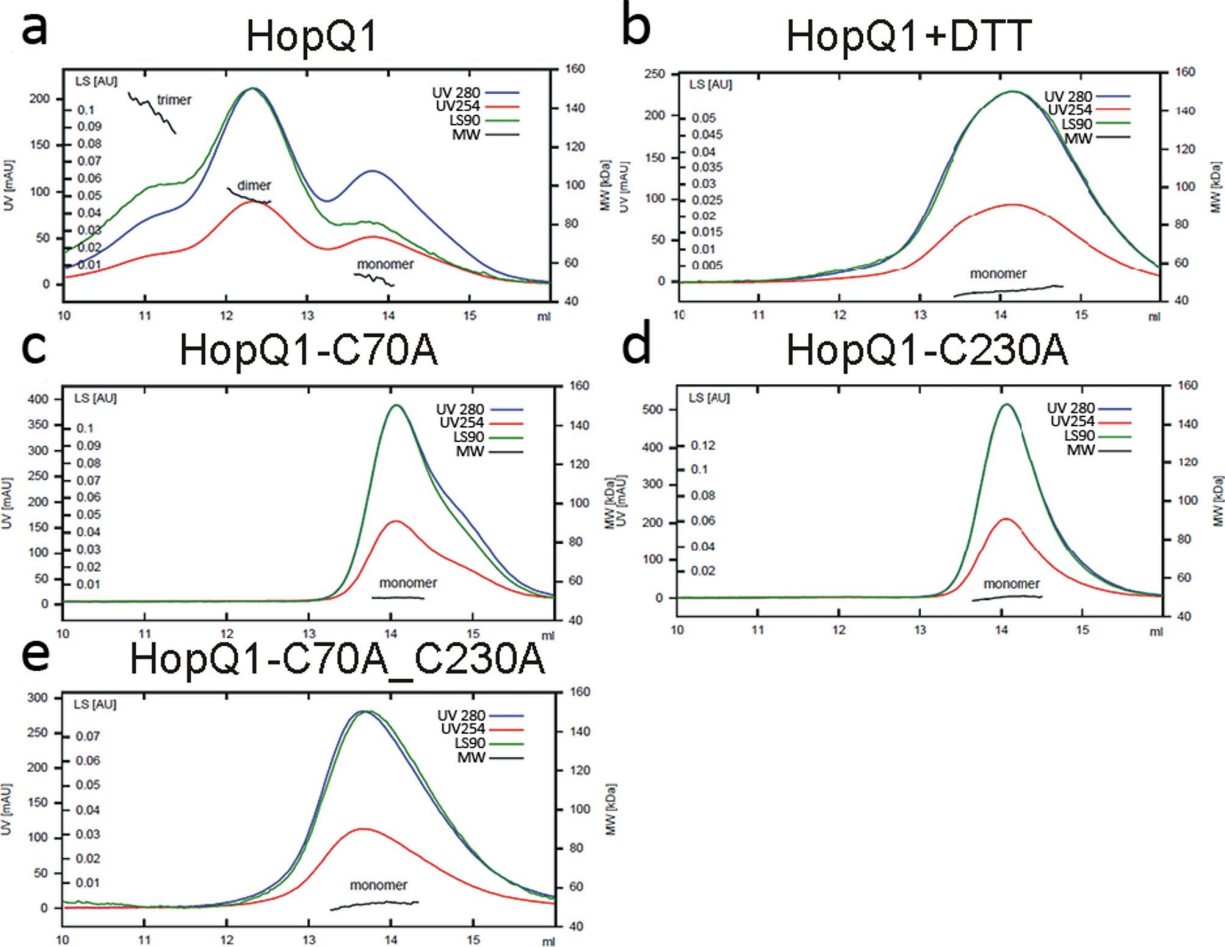
Disulfide-linked HopQ1 oligomers. Recombinant HopQ1 with a C-terminal 6 × His epitope was subjected to gel filtration coupled to MALS analysis under native (**a**) or reducing (**b**) conditions. The cysteine single and double mutants C70A (**c**), C230A (**d**), and C70A_C230A (**e**) were analyzed under native conditions. Blue and red traces correspond to absorbance at 280 nm and 254 nm, respectively; green trace indicates static light scattering at 90° (LS90), and black indicates molecular weights. The derived molar masses for the monomer, dimer, and trimer are 52 kDa ± 2%, 94 kDa ± 1%, and 138 kDa ± 1%, respectively (the theoretical masses for the monomer, dimer, and trimer are 49.8, 99.6, and 149.4 kDa, respectively).

We performed size exclusion chromatography (SEC) coupled with multi-angle light scattering (MALS) analysis of HopQ1. This technique allows the determination of molecular mass of proteins, that are separated based on their retention. As shown in Fig. 1a, SEC-MALS analysis of HopQ1 tagged with 6 × His revealed that the recombinant protein purified from *Escherichia coli* eluted as a monomer, dimer, and trimer. HopQ1 contains two conserved cysteines (C70 and C230), which could be involved in oligomer formation. Consistently, treatment with 5 mM dithiothreitol (DTT), a reducing agent, converted HopQ1 oligomers into monomers (Fig. 1b). In parallel, we mutated either one or both cysteines into an alanine, and again subjected the recombinant proteins to SEC-MALS analyses (Fig. 1c–e). Under these conditions, the mutated HopQ1 variants were detected only as monomers. These results indicate that the HopQ1 oligomeric state depends on disulfide bridge formation.

**Fig. 2:**
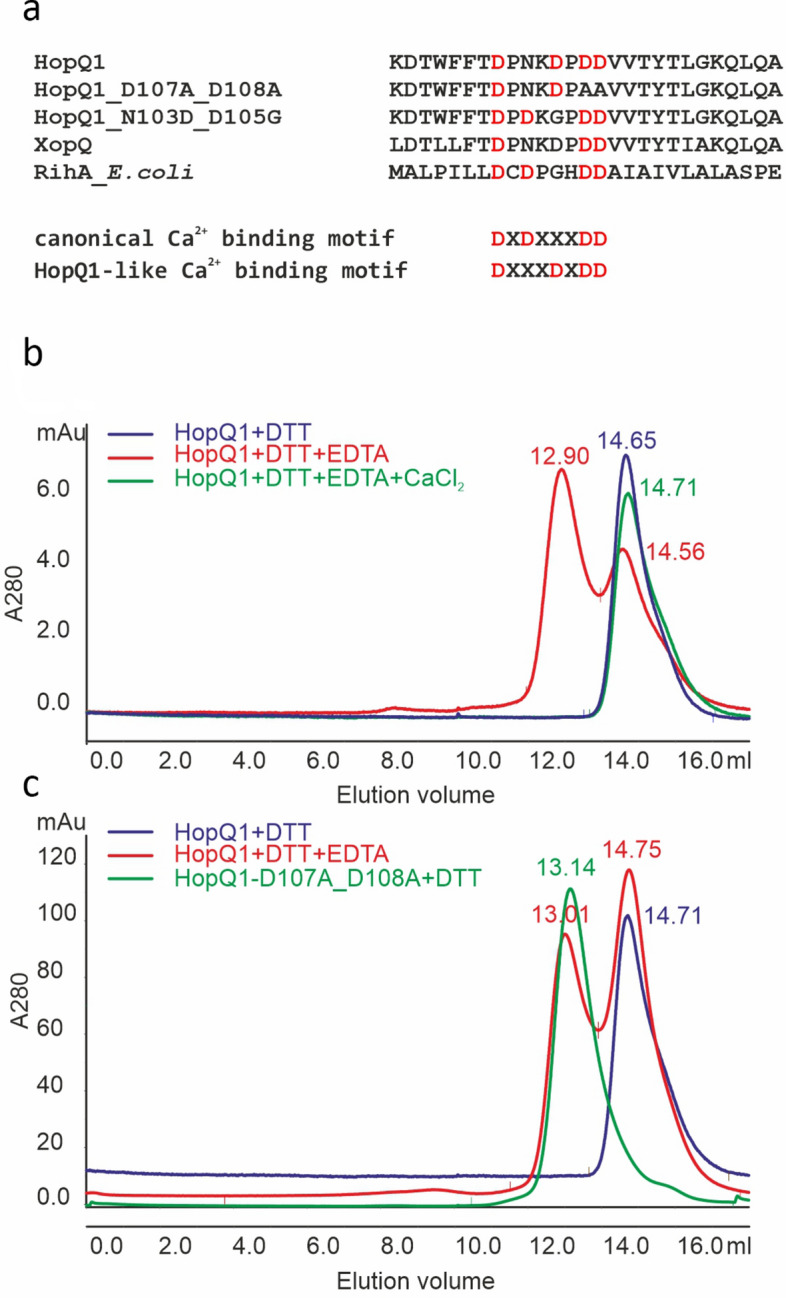
Depletion of calcium ions induces HopQ1 dimer formation in the presence of the reducing agent. Representative gel filtration runs on a Superdex 200 column are shown. (**a**) Comparison of canonical and HopQ1-like Ca^2+^ binding motifs (**b**) Blue trace, recombinant HopQ1 with a C-terminal 6 × His epitope was eluted under reducing conditions (5 mM DTT) as a monomer (elution volume of 14.65 ml). Red trace, on application of chelating agent (1 mM EDTA), HopQ1 was eluted as two peaks corresponding to monomer (elution volume of 14.56 ml) and dimer (elution volume of 12.90 ml). Green trace, on addition of chelating agent and calcium ions (1mM EDTA, 2 mM CaCl_2_), HopQ1 eluted only as monomers (elution volumes of 14.71 ml). (**c**) Green trace, the HopQ1 mutant in the calcium-binding site was eluted only as dimers (peak 13.14 ml). As a control, HopQ1 was used under reducing conditions (blue trace, monomer, elution volume of 14.71 ml) or upon application of chelating agent (red trace; monomer and dimer with elution volumes of 14.75 and 13.01 ml, respectively). Prior to gel filtration the samples were analyzed by immunodetection (Fig. S1a, b). The experiment was performed twice with similar results.

HopQ1 and its homologs, including XopQ from *Xanthomonas* spp., possess an atypical calcium ion binding motif in the predicted catalytic center (Fig. 2a). Although its sequence differs from that present in the canonical Nucleoside Hydrolases (NHs), structural studies of XopQ from *Xanthomonas oryzae*, showed that this site indeed coordinates calcium ion, and its location within the protein structure is reminiscent of the classical NH^8^. To test the effect of calcium on the HopQ1 structure, we added to the reduced form of the protein either a chelating agent or calcium ions, and subsequently subjected the samples to gel filtration (Fig. 2b). The results showed that HopQ1 self-associated in the presence of 1 mM EDTA. This process was reversible, because the addition of 2 mM calcium resulted in HopQ1 monomeric structures. In contrast, the chelating agent did not affect the structure of RihA, a pyrimidine nucleoside hydrolase from *E. coli* (also known as YbeK), containing a canonical calcium binding site^5,9^, that in the presence of 1 mM EDTA was eluted as a tetramer (Fig. S2). Next, we analyzed HopQ1-D107A_D108A, a variant mutated in two aspartic acids of HopQ1^10^. D108 corresponds to one of the three aspartate residues involved in coordination of calcium ion in the structure of Inosine-Uridine Nucleoside N-Ribohydrolase of *Crithidia fasciculata*^11^. HopQ1-D107A_D108A was subjected to gel filtration, which revealed that the mutant existed only as dimers under all the conditions tested (Fig. 2c). Collectively, these results support the model that reversible calcium binding to the HopQ1 catalytic center modulates the equilibrium between monomeric and dimeric states of the effector.

### Stability of HopQ1 depends on both pH and [Ca^2+^]

**Fig. 3:**
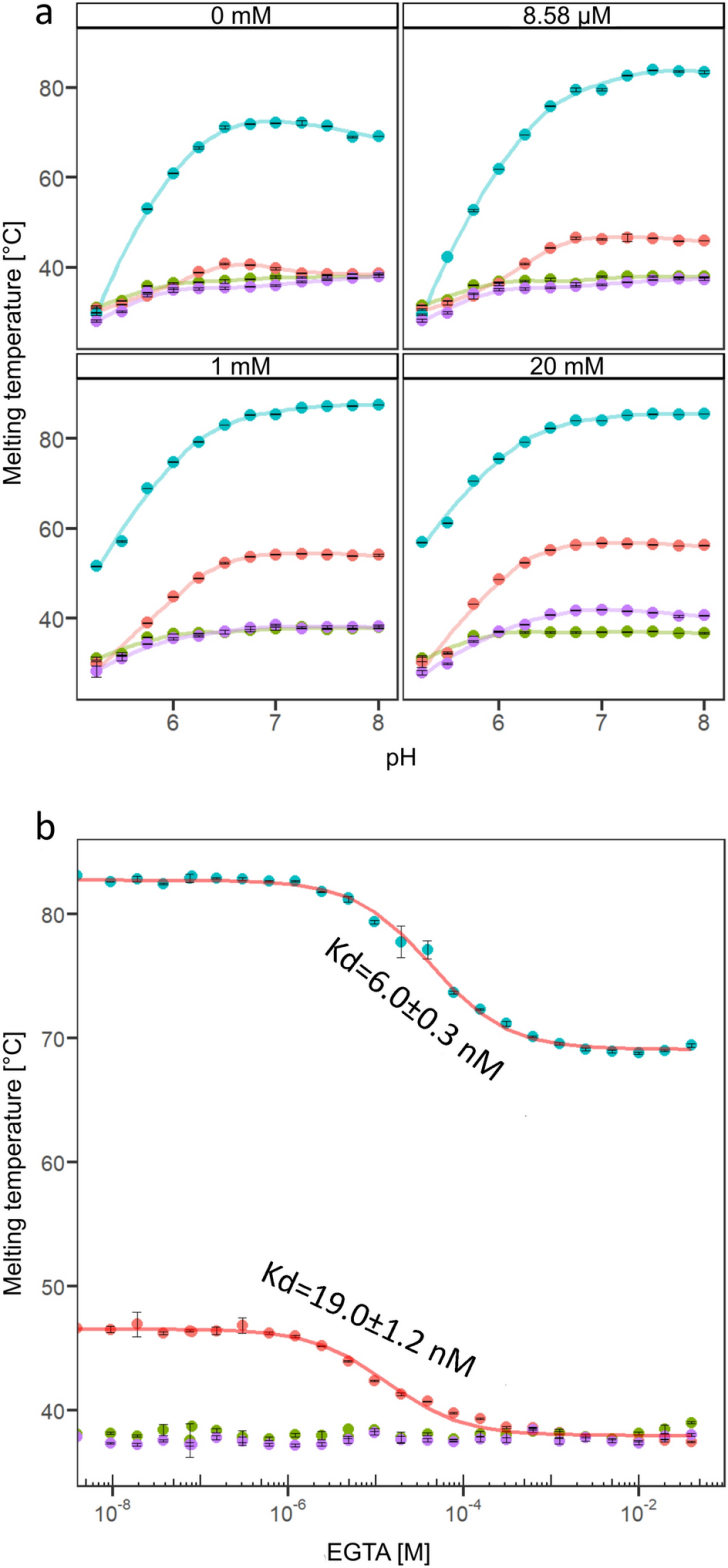
Thermal stability of 6 × HIS-tagged proteins: HopQ1-WT (red), HopQ1-D107A_D108A (green), HopQ1-N103D_D105G (purple) and RihA (blue). RihA is a pyrimidine nucleoside hydrolase, containing a canonical calcium binding motif, and was used as a control. (**a**) Relationships between protein melting temperature, pH, and free Ca^2+^ concentration (0 mM—presence of 200 µM EGTA; 8.58 µM—retroactively-estimated calcium concentration in the absence of EGTA, see Figs. S3–S4). For pH adjustment MES (pH ≤ 6.75) and HEPES (pH ≥ 6.75) buffers were used. Lines correspond to local regression (LOESS). (**b**) The data obtained after at increasing EGTA concentrations. The experiment was performed twice with similar results.

To get more insight into HopQ1-Ca^2+^ interaction, a series of experiments were carried out using nano differential scanning fluorimetry (nanoDSF). We measured melting temperatures (T_m_) of the wild type HopQ1 supplemented with various Ca^2+^ concentrations at pH values corresponding to the physiological conditions (Fig. 3a). These analyses revealed that T_m_ of HopQ1 was significantly increasing along with the elevated pH in the presence of Ca^2+^. In turn, stability of RihA was also rising with pH, but its melting temperature was in general much higher than of HopQ1 and only at very low concentration (up to 8.58 µM) dependent on Ca^2+^. In contrast, HopQ1-D107A_D108A, the variant of the effector with substitutions abrogating Ca^2+^ binding, showed very low stability corroborating the importance of Ca^2+^ for HopQ1 structure maintenance. Similarly, HopQ1-N103D_D105G displayed a low stability under all conditions used suggesting that while we had reconstructed the canonical linear Ca^2+^ binding sequence (Fig. 2a) we failed to obtain a functional binding site. Inspection of the modelled HopQ1 structure demonstrates that N103D strongly destabilizes the Inosine-Uridine Nucleoside N-Ribohydrolase fold by placing the charged D103 sidechain in the non-polar environment. At the same time, D105G replacement enables the K104 sidechain to compete with Ca^2+^ for the binding site.

We added increasing concentrations of calcium to the samples to estimate the strength of Ca^2+^ binding to HopQ1. The K_d_ values inferred from this experiment were at a low millimolar range, questioning a role of calcium in stabilization of HopQ1 structure in vivo, and suggests the presence of nonspecific calcium binding sites (Fig. S3). The addition of EGTA at increasing concentration reduced, however, the stability of the wild-type HopQ1 and RihA. At the same time, no such effects were observed for HopQ1-D107A_D108A and HopQ1-N103D_D105G (Fig. 3b). These data showcase the ability of HopQ1 and RihA to bind calcium in a low nanomolar range, implying that both proteins were saturated at a residual calcium concentration in the sample (estimated to 8.6 µM). Collectively, HopQ1 was most stable under neutral conditions, that mimic those of the host cytoplasm. In contrast HopQ1-D107A_D108A variant displayed a greatly reduced stability under all the conditions studied and remained largely insensitive to the changes of experimental cues (Fig. 3). These results not only point at the pH driven changes in the affinity for calcium, but also show that the pH itself can modulate the stability of HopQ1.

### Elimination of Ca^2+^ binding variant promotes dimerization of HopQ1 in planta

**Fig. 4:**
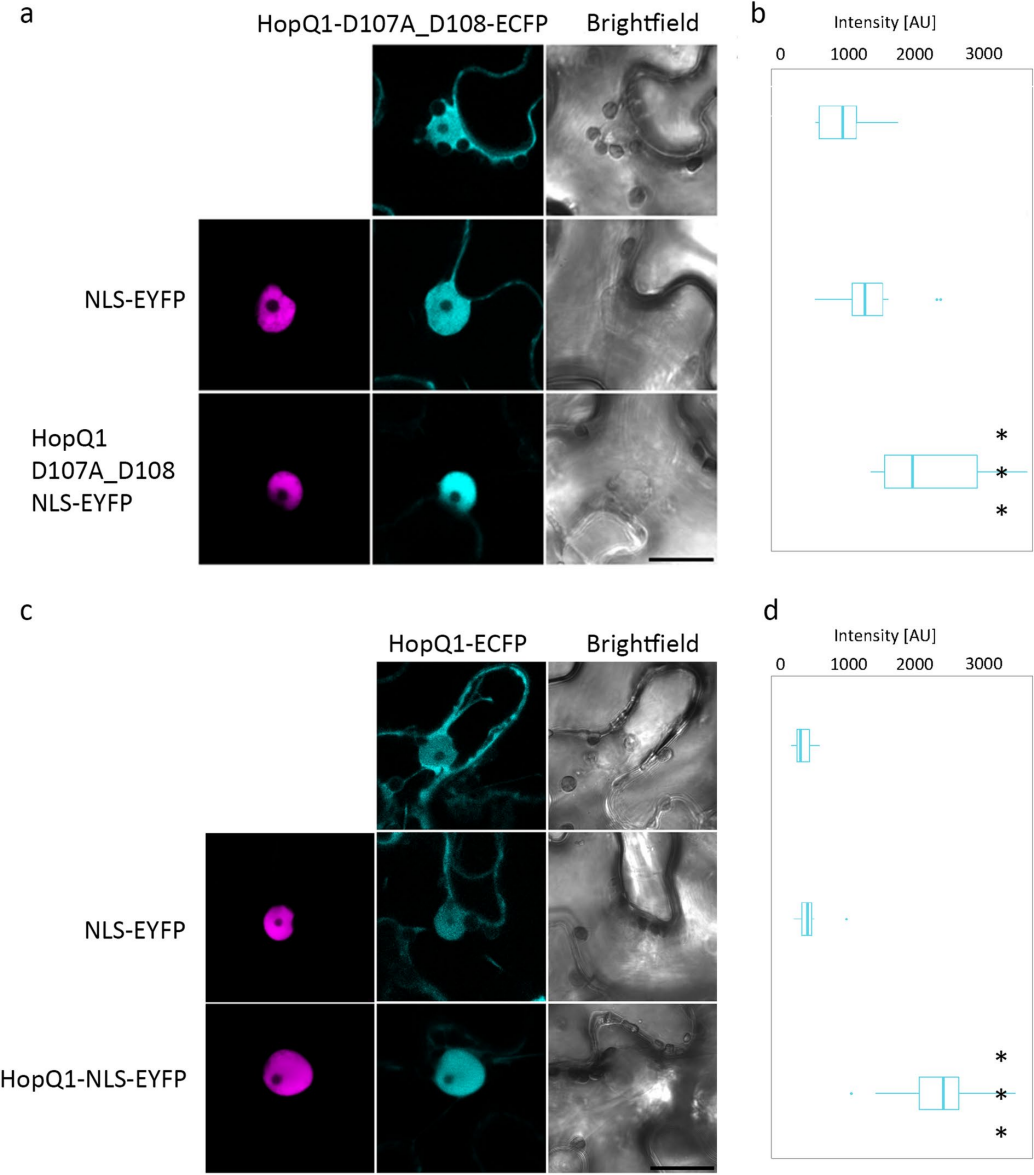
HopQ1 forms dimers in planta. Confocal images show representative *N.* *benthamiana* leaf epidermal cells transiently co-expressing pairs of HopQ1 (**a**) or HopQ1-D107-D108A (**b**) fused to ECFP along with the same variants fused to EYFP and nuclear localization signal (NLS). The photographs were taken 72 h after agroinfiltration. For each variant, approximately 20 transformed cells were examined and fluorescence intensity of ECFP was measured in each nucleus (**c**, **d**). Bars = 10 μm. Asterisks indicate significantly higher ECFP intensity, when compared to expression of single HopQ1 (**a**) or HopQ1-D107-D108A (**b**) fused to ECFP, *p*-value < 0.001 assessed by Wilcoxon test. The expression levels of proteins used in the experiment was analyzed by immunodetection (Fig. S5).

To assess whether HopQ1 self-assembles in vivo, we performed fluorescence resonance energy transfer (FRET) analysis (Table 1). FRET was measured using fluorescence lifetime imaging (FLIM) of HopQ1 variants fused to enhanced cyan fluorescent protein (ECFP), which were co-expressed in *N. benthamiana* along with the same variant fused to enhanced YFP (EYFP). As positive and negative controls, we analyzed the association of 14-3-3a, with HopQ1 and HopQ1-S51A, respectively^12^. In agreement with the biochemical data, FRET, measured as a decreased ECFP fluorescent lifetime, was observed between HopQ1-D107A_D108A molecules. In contrast, we did not detected any reduction in the ECFP fluorescent lifetime for the wild-type HopQ1. FRET occurs between fluorescent tags when the distance between them is less than 10 nm^13^. Our results indicate that HopQ1-D107A_D108A molecules were close enough to each other for FRET process to occur, suggesting their different spatial arrangement, compared to the wild-type proteins. This finding, however, does not exclude that the wild-type HopQ1 forms oligomers in planta. To test this possibility, we employed a MILo (Modification of Intra-cellular Localization) method, a variant of the nuclear transport assay^14,15^ which is not limited by the distance between tags of the interacting proteins^16^. This technique is based on an assumption, that co-expression of two interacting proteins showing distinct localization patterns affects their reciprocal distributions. To obtain effector variants confined to the nucleus, we added a strong nuclear localization signal (NLS) to their fusion with EYFP. Subsequently, we checked their putative effect on the respective HopQ1 variants fused to ECFP. In line with the FRET data, HopQ1-D107A_D108A-ECFP variant was shifted into the nucleus compartment in the presence of HopQ1-D107A_D108A with the forced nuclear localization (Fig. 4a,b). Interestingly, co-expression of HopQ1-ECFP along with the nucleus confined form, that is HopQ1-EYFP-NLS, shifted it to the nucleus (Fig. 4c,d). This observation suggests that the wild-type HopQ1 self-assembles despite the intracellular calcium concentration^17^ would indicate the effector is in the calcium-bound form.

**Table 1:** FLIM-FRET analysis indicates that calcium-free HopQ1 may associate in plant cells.

Donor	Acceptor	τD (ns ± SD)^a^	τDA (ns ± SD)^b^	FRET efficiency^c^
HopQ1-ECFP	HopQ1-EYFP	2.96 ± 0.02	2.94 ± 0.02	1.0
HopQ1-D107A_D108A-ECFP	HopQ1-107A_D108A-EYFP	2.83 ± 0.05	2.57 ± 0.03	9.2
Nt14-3-3a-ECFP^d^	HopQ1-EYFP	2.96 ± 0.01	2.54 ± 0.01	14.2
Nt14-3-3a-ECFP	HopQ1-S51A-EYFP	2.96 ± 0.01	2.97 ± 0.01	–

^a^τD, mean donor lifetimes in the absence of the acceptor ± SD.

^b^τDA, mean donor lifetimes in the presence of the acceptor ± SD.

^c^FRET efficiency = [1—(τDA/τD)] × 100%

^d^ interaction of Nt14-3-3a with the wild-type HopQ1 or HopQ1-S51A were used as positive and negative control, respectively.

Collectively, these data corroborate the model that HopQ1 forms higher order structures in planta suggesting that structural changes, possibly induced by local calcium depletion, facilitate this process. Our earlier analysis showed that the nuclear trafficking of HopQ1-D107A_D108A is faster compared to the wild-type effector^10^. In line with that observations, HopQ1-D107A_D108A showed slightly enhanced nuclear localization compared to the wild-type HopQ1 (Fig. S6). This led us to hypothesize that calcium accessibility is another mean to control nucleocytoplasmic distribution of the effector.

## Discussion

How type three effectors enter injectisomes and reach their final destination is an important aspect of the gram-negative bacteria pathogenesis. This process starts with penetration of intrinsically disordered N-termini of the effectors into the channel of TTSS export apparatus. Further translocation, that is movement into the TTSS needle (in phytopathogenic bacteria referred to as pilus) requires unfolding of the effectors’ globular domains^18^. This process is facilitated by specialized chaperones that are supposed to maintain effectors in the unfolded state^19^. To the unfolding as well as to the release of the chaperones from the TTSS substrates contributes ATPase, a member of YScN family, that provides also energy for the translocation. There are, however, exceptions to this general model. It has been shown that in some bacterial species the presence of the functional ATPase is not critical for the translocation^20^. Similarly, the chaperones are not always employed by the bacteria for all the substrates. In *P. syringae*, for instance, majority of the effectors is chaperone-independent^3^.

Until recently, it has been broadly accepted that effectors have low thermodynamic stability, and this feature enables them efficient unfolding prior to the entrance to TTSS^21,22^. Studies on AvrPto from *P. syringae* led to a proposal of a comprehensive model describing, how the effector undergoes consecutive unfolding and folding, while facing changes in pH values in the bacterial cytosol, TTSS conduit, and within plant cell^7,23^. Recently however, an alternative unfolding mechanism has been proposed for SptP and SopE2. These two *Salmonella* effectors are thermodynamically stable, but they display low resistance to mechanical pulling^24^. Thus, it was hypothesized that they may undergo unthreading while passing through TTSS conduit. In line with this model, structural studies of an active needle complex of *Salmonella enterica* engaged with SptP effector suggested that SptP might be transported in a nonglobular state containing α-helices, without further unfolding^2^.

Our analyses show that thermal stability of HopQ1, another *P. syringae* type three effector changes in the pH range the effector encounters while traversing from prokaryotic to eukaryotic cell. Additionally, calcium plays an important role in stabilizing HopQ1 structure. In the absence of calcium, HopQ1 displays relatively low thermal stability (T_m_ ~ 40 °C). Along with the rising calcium concentration the thermal stability of the effector significantly increases by more than 10 °C. Consistent with this model, HopQ1-D107A_D108A, the HopQ1 variant mutated to eliminate calcium binding, is thermolabile in the analyzed pH range (Fig. 3).

The relatively high T_m_ of HopQ1 at the neutral pH in the calcium presence, compared to the T_m_ under the slightly acidic conditions, implies that intrinsic properties of HopQ1 may not only facilitate pH-dependent unfolding of the effector prior to the entrance to injectisome, but also efficient refolding upon calcium binding at the neutral or slightly alkaline pH of the host cell. Thus, the atypical calcium binding motif may enable also self-maturation of HopQ1. In contrast, other bacterial effectors often co-opt host cellular machinery to acquire mature conformation. AvrRpt2 from *P. syringae* binds plant cyclophilin, which catalyzes prolyl isomerization inducing proper folding and self-cleavage of the effector^25^. In turn, HopBF1 of *P. syringae* mimicking the client binds to the host HSP90 chaperone, which facilitates final maturation and activation of the effector^26^.

Self-assembly appears to be a means fine-tuning HopQ1 subcellular distribution. We observed that HopQ1 formed higher order structures in vitro. This process was promoted by non-reducing conditions or calcium depletion. Co-expression of HopQ1 with its nucleus-localized form shifted the effector to the nucleus suggesting an interaction between both variants. Employing FRET measurement we could not observe formation of the wild -type complexes in planta but we did observe association of HopQ1-D107A_D108A variants (Table 1). This indicates that HopQ1-D107A_D108A displaying lower thermal stability than wild-type HopQ1 is more prone to form complexes or aggregates in planta. Thus, we can assume that calcium availability may control self-association of the effector in host cells. The experiment in which we co-expressed the wild-type HopQ1 along with the variant with forced nuclear localization let us to speculate that such complexes might be possibly formed under specific conditions. Since local calcium dynamics may be modulated by calcium-binding proteins we analyzed interactors of HopQ1 variants. We found that wild-type HopQ1, HopQ1 variant unable to bind calcium (HopQ1-D107A_D108A) and the nucleus confined variant (HopQ1-NLS) possess specific repertoires of interactors. The fact that among the interactors of HopQ1 are proteins regulating calcium levels provides a putative mechanism that may contribute to oligomerization of the effector in the host cells (Fig. S7), but this hypothesis requires experimental verification.

Our previous experiments showed that HopQ1-D107A_D108A displayed an accelerated trafficking towards the nucleus compared to the wild-type HopQ1^10^. This suggests that the assembly of HopQ1 complexes may promote nuclear distribution of the effector. Compared with classic nucleoside hydrolases, the XopQ structure lacks two antiparallel β-strands close to the catalytic center, which are involved in intersubunit interactions. Due to this change, the catalytic loop adopts a different conformation^8^. Here we propose a model in which the structural changes in HopQ1-like proteins (compared with those in classical NHs) enable reversible regulation of their oligomeric state in a redox- and calcium-dependent manner. The formation of HopQ1 oligomers under non-reducing conditions in the presence of calcium suggests that these complexes consist of protomers coordinating calcium ions, reminiscent of the XopQ structure containing calcium ion, resolved by crystalography^8,27^. In contrast, decreased thermal stability of HopQ1-D107A_D108A suggests that this variant self-associates or aggregates in the partially unfolded state. Our in silico analysis additionally supports the mechanism described above. The putative structures of HopQ1 dimers were modelled with the aid of AlphaFold (Fig. S8). The resulting models can be clustered in two orientations of the symmetric dimeric form, one of which resembled the dimer identified in XopQ structure complexed with ADP (PDB 4P5F 10.2210/pdb4P5F/pdb)^8^. Interestingly, in the other one, the interface includes the regions proximal to the calcium-binding residues. This suggests that assembly of such dimers may be coupled with Ca^2+^ binding, and that such an alternative interface for dimerization may facilitate the formation of higher-order oligomers.

In summary, our results deliver new details into the working model of HopQ1 dynamics. We propose that the atypical calcium binding motif of HopQ1 provides environmentally driven control mechanism that may enable trafficking of the effector from the bacterial cytosol to the host cell in a chaperone independent manner, and its refolding once the effector reaches the host cell. This process is followed by phosphorylation of HopQ1 possibly carried out by kinases of the CDPK/SnRK superfamily^12^ that activity is also controlled by the intracellular calcium level. The modification of HopQ1, in turn, is a prerequisite for plant 14-3-3s binding and maintenance of its proper partitioning within the host cell. The nucleocytoplasmic partitioning of the effector seems to be affected also by formation of higher order structures that may occur due to local decrease in calcium concentration and/or an appropriate redox state. This suggests that the mechanisms controlling effector distribution may react to changing cellular conditions during the course of infection.

Noteworthy, intracellular calcium concentration in both prokaryotes and eukaryotes oscillates in high nanomolar ranges^17,28^. Similarly to plants, phytopathogenic bacteria were also shown to produce transients in [Ca^2+^] in response to environmental cues. Moreover, an increase in [Ca^2+^] level promotes bacterial virulence, regulating among other biofilm formation and the TTSS machinery^29,30^. Taken together, phytopathogenic bacteria adapted their virulence strategy to host calcium concentration^31^ which results in the proper level and structure of effectors involved in tissue colonization.

## Methods

### Protein expression and purification

Recombinant HopQ1 (AAZ37975.1) from *Pseudomonas savastanoi* pv. *phaseolicola* 1448A (former *Pseudomonas syringae* pv. *phaseolicola* 1448A) its variants were produced in *Escherichia coli* BL21 Rosetta in the C-terminal fusion with 6 × His, and purified by nickel-affinity chromatography and ion-exchange chromatography (Q-Sepharose column; GE Healthcare, Little Chalfont, Buckinghamshire, United Kingdom) as described previously^12^.

### Site-directed mutagenesis

To create HopQ1-N103D_D105G, HopQ1-C70A, HopQ1-C230A, HopQ1-C70A_C230A variants, target plasmid carrying *hopQ1* was PCR amplified with two appropriate phosphorylated primers (Table S1). The primers were designed so that they annealed back-to-back with the plasmid, and one primer carried the desired mutations. Following amplification with Phusion Hot Start II DNA Polymerase (Thermo Fisher Scientific; Waltham, MA, USA), templates were removed by digestion with DpnI (Thermo Fisher Scientific), and mutated PCR products were circularized with T4 DNA ligase (Thermo Fisher Scientific). The resulting constructs were transformed into *E. coli* DH5α. The plasmids isolated from the clones selected by colony PCR were sequenced.

### Constructs for subcellular localization experiments

The construction of wild-type *hopQ1-EYFP*, *hopQ1-S51A-EYFP* in pGWB441, and *Nt14-3-3a-ECFP* in pGWB444 was described previously^12^. The *hopQ1-D107A_D108A* sequence was cloned into the pENTR/D-TOPO vector using the same PCR primers^10^. The reverse primer was without the native stop codon for HopQ1-D107A_D108A C-terminal fusion. To generate the expression clone, LR recombination was performed using the entry clone obtained and one of the pGWB destination vectors 441 or 444^32^. To construct Gateway binary vector containing nuclear localization signal (NLS) fused to EYFP, the sequences encoding NLS were PCR amplified with primers that contained AfeI restriction sites, cloned into pJET1.2, and re-cloned into the binary vector pGWB414 upstream of *EYFP*. The resulting plasmid were used for LR recombination. To generate the *hopQ1* and *hopQ1-D107A_D108A-NLS-EYFP* constructs, the entry clones were LR recombined into the modified pGWB414 vector containing the NLS.

### Confocal microscopy

*Agrobacterium*-mediated transient expression of HopQ1 variants fused to EYFP and ECFP in *Nicotiana benthamiana* leaves was used to determine a subcellular localization. Bacterial suspensions of GV3101 strain in MES buffer [10 mM MgCl_2_, 10 mM MES pH 5.6, 150 µM acetosyringone] were infiltrated into 5-week old plants then section of leaves were inspected 72 h post infiltration. The results were evaluated using a Nikon C1 confocal system built on TE2000E and equipped with a 60 × Plan-Apochromat oil immersion objective (Nikon Instruments B.V. Europe, Amsterdam, Netherlands). EYFP fusion proteins were excited with a Sapphire 488 nm laser (Coherent, Santa Clara, CA, USA) and observed using the 515/530 emission filter. ECFP fusion proteins were excited with a 408 nm diode laser and detected using the 460/50 emission filter. For publication, single optical sections with distinctly visible nucleoli were selected to ensure that similar focal planes were compared for all tested variants. Quantification of fluorescence intensities in the nuclear and cytoplasmic regions was performed using ImageJ software^33^. Data analyses were carried out in R (http://www.r-project.org).

For FLIM-FRET analyses, proteins of interest were fused to ECFP (donor) and transiently expressed in *N. benthamiana* leaves in the presence and absence of the potential interacting partner fused to EYFP (acceptor). Cells were imaged with a FV100 confocal system (Olympus) equipped with a 60 × water immersion objective lens. For FLIM, ECFP fusion proteins were excited with a 440 nm pulsed diode laser (Sepia II, PicoQuant, Berlin, Germany) and detected using a 482/35 bandpass filter. Images were acquired with a frame size of 256 × 256 pixels. Photons were collected with a SPAD detector and counted with the PicoHarp 300 TCSPC module (PicoQuant). The obtained data were analyzed with Symphotime software (PicoQuant). Fluorescence lifetimes of CFP in nuclei were calculated by fitting a bi-exponential decay model.

For FRET analysis, the fluorescence lifetime of the donor/acceptor pair (τ DA) was compared with that of donor alone (τ D). The FRET efficiency as calculated as E = (1 − τ DA/τ D) × 100%, where τ D is the fluorescence lifetime of a donor in the absence of an acceptor and τ DA that of a donor in the presence of an acceptor. Finally, eight to ten nuclei per combination were analyzed and the average of the values was taken for analysis. Interaction of Nt14-3-3a with the wild-type HopQ1 or HopQ1-S51A (the variant with eliminated 14-3-3 binding site) were used as positive and negative control, respectively^10,12^.

### MALS analysis

For MALS analysis, the samples were separated using a Superdex 200 10/300GL column in buffer containing 100 mM Tris–HCl, 150 mM NaCl, 5 mM DTT, pH 8.0 at 4 °C. ). 0.4 mg of the protein was used for each run. Preincubation with EDTA was used to remove free Ca^2+^ ions, then CaCl_2_ was added where indicated. The analysis was performed using the Wyatt Dawn Heleos II apparatus connected to Optilab T-rex (Wyatt Technologies, Santa Barbara, CA, USA) supported with a refractometer. Results were analyzed using Astra 6 software according to the manufacturer’s instructions.

### Thermal shift assay analysis

C-terminally 6 × HIS-tagged wild-type HopQ1 protein, HopQ1-D107A_D108A and RihA, a pyrimidine-specific ribonucleoside hydrolase from *E. coli* K-12 were expressed in *E. coli* BL21 Rosetta strain. Recombinant proteins were purified and transferred to an appropriate to pH buffer HEPES (50 mM HEPES, 150 mM NaCl pH 7.00) or MES (50 mM MES, 150 mM NaCl, pH 5.50 or 6.25) containing 10 × SYPRO Orange (Thermo Fisher Scientific). Final protein concentration in analyzed samples was 5 µg/ml. Protein samples were incubated on ice for 10 min and then loaded to nanoDSF Grade Standard Capillaries and scanned with Prometheus NT.48 Series (Nanotemper, Munich, Germany). Excitation power was set to 70%, temperature range was 20–80 °C with a slope of 1 °C per minute.

To analyze the high Ca^2+^ concentration effect, prior to TSA the purified protein samples were supplemented with appropriate amounts of CaCl_2_ and EGTA in accordance with calculations performed in MAXCHELATOR software in order to determine free Ca^2+^ in samples (https://somapp.ucdmc.ucdavis.edu/pharmacology/bers/maxchelator/CaEGTA-TS.htm).

Ca^2+^ chelation experiments were performed with a series of 24 twofold dilutions of EGTA without Ca^2+^ supplementation. Initial Ca^2+^ concentration in samples was estimated retroactively (Fig. S3) and was roughly equal to protein concentration.

K_d_ values were determined from experimental data (see Fig. S4) using equation:

$$T_{m} = T_{0} - \frac{{dT \times \left[ {EGTA} \right]}}{{\left[ {EGTA} \right] + K_{i} \times \left( {1 + \frac{{\left[ {Ca} \right]}}{{K_{d} }}} \right)}}$$

*Tm*—melting temperature; *T0*—melting temperature in the absence of EGTA; K_d_ value for HopQ1. *dT*—EGTA-induced decrease in the melting temperature (*dT* ≈ T_m,holo_ − T_m,apo_). [EGTA]—EGTA concentration in M. [Ca]—total Ca^2+^ concentration in M. *K*_*i*_—Ca^2+^-binding affinity for EGTA (29.6 nM). *K*_*d*_—Ca^2+^-binding affinity for protein of interest.

## Supplementary Information

Supplementary Material.

## Data Availability

The data that support the findings of this study are available from the corresponding author on reasonable request.
