# Supplementary material for: Noncanonical calcium binding motif controls folding of HopQ1, a Pseudomonas syringae type III secretion effector, in a pH-dependent manner
Source: Sci Rep. 2024 Dec 30;14:31796. doi: 10.1038/s41598-024-82848-z (PMC11685791; doi:10.1038/s41598-024-82848-z)

Supplementary Information

Noncanonical calcium binding motif controls folding of HopQ1, a *Pseudomonas syringae* type III secretion effector, in a pH-dependent manner

Fabian Giska[1], Wojciech Rymaszewski[1], Malgorzata Lichocka[1], Marcin Piechocki[1], Jakub Kwiatkowski[1], Jarosław Poznański[1], Magdalena Górecka[1], Magdalena Krzymowska[1]

Short title: Calcium binding motif controls HopQ1 folding

[1]Institute of Biochemistry and Biophysics, Polish Academy of Sciences, Warsaw, Poland

A

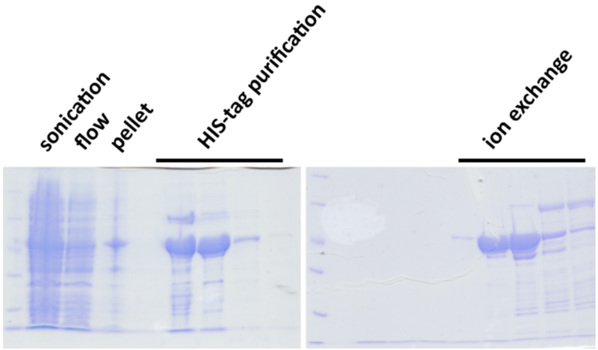

B

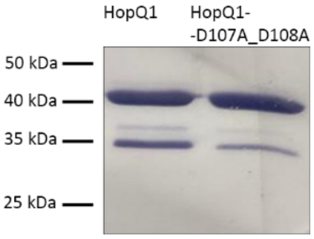

Supplementary Figure S1 The purity of samples tested was monitored at the various steps of purification pipeline with the use of SDS-PAGE (in 12% gel) followed by Coomassie staining (A) or immunodetection with the use of anti-His mouse antibodies (dilution 1:2000) (B). Source data is provided below.

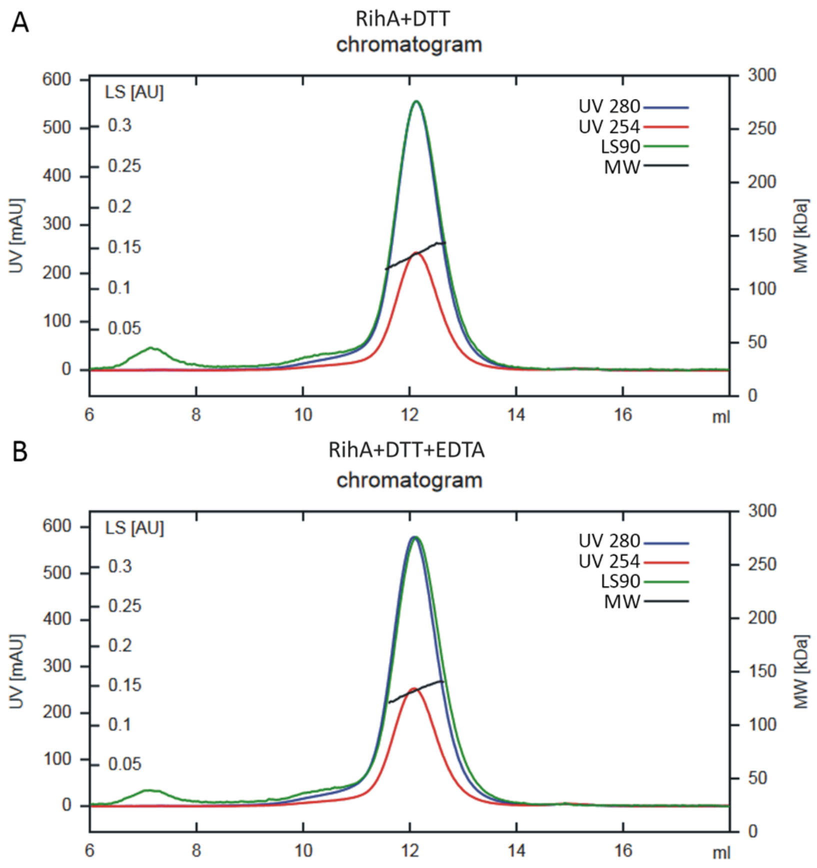

Supplementary Figure S2 RihA forms tetramers independent of the presence of chelating agent. Recombinant RihA with a C-terminal 6xHis epitope was subjected to gel filtration coupled to MALS analysis under reducing conditions in the absence (A) or presence of the chelating agent (B). Blue and red traces correspond to absorption at 280 nm and 254 nm, respectively; green trace indicates static light scattering at 90° (LS90), and black indicates molecular weights. The derived molar masses were 133 kDa (+/- 1%) for RihA + 5 mM DTT and 132 kDa (+/- 1%) for RihA + 5 mM DTT and 1 mM EDTA, whereas the theoretical masses are 34.888 kDa for the monomer and 139.552 kDa for the tetramer.

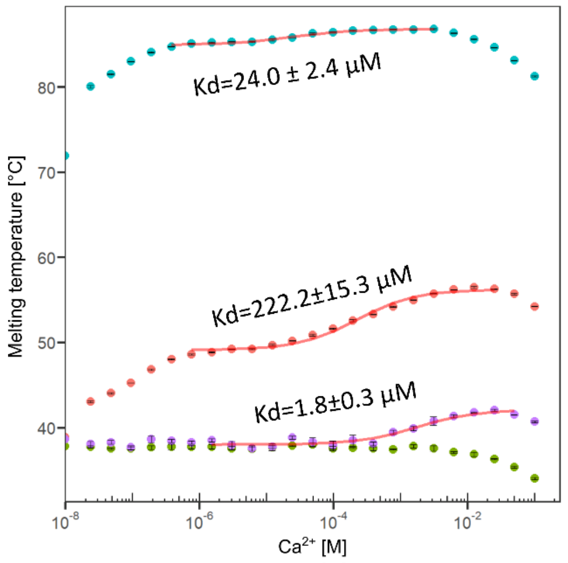

Supplementary Figure S3 Relationships between protein melting temperatures and free Ca^{2+} concentration (adjusted with EGTA and $CaCl_2$ based on MAXCHELATOR calculations). HopQ1-WT (red), HopQ1-D107A_D108A (green), HopQ1-N103D_D105G (purple) and RihA (blue). Error bars are ±SD. Curves were fitted, as described in Fig. 3b. In this experiment, the binding affinity of a weak, unspecific site was monitored, while the strong one was predominantly saturated under all sampled conditions.

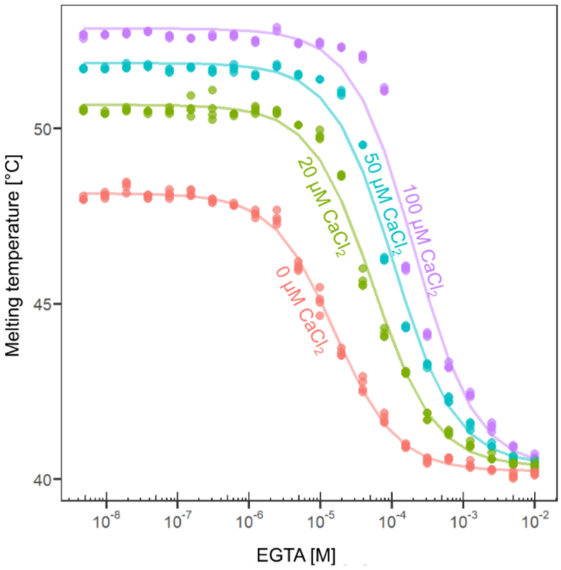

Supplementary Figure S4 EGTA pseudotitration of the C-terminally 6×HIS-tagged wild-type HopQ1 protein in the presence of different concentrations of externally added $CaCl_2$ for retroactive estimation of initial Ca^{2+} concentration. Curve fitting was done setting initial calcium concentration as a variable between 0 and 20 µM in 10 nM increments (2000 fits per series). The value that gave the best fit for all 4 externally-added calcium concentrations (lowest standard deviation from all series), i.e. 8.58 µM was considered the best estimation of initial Ca^{2+} concentration in all samples and thus used as a constant in further calculations.

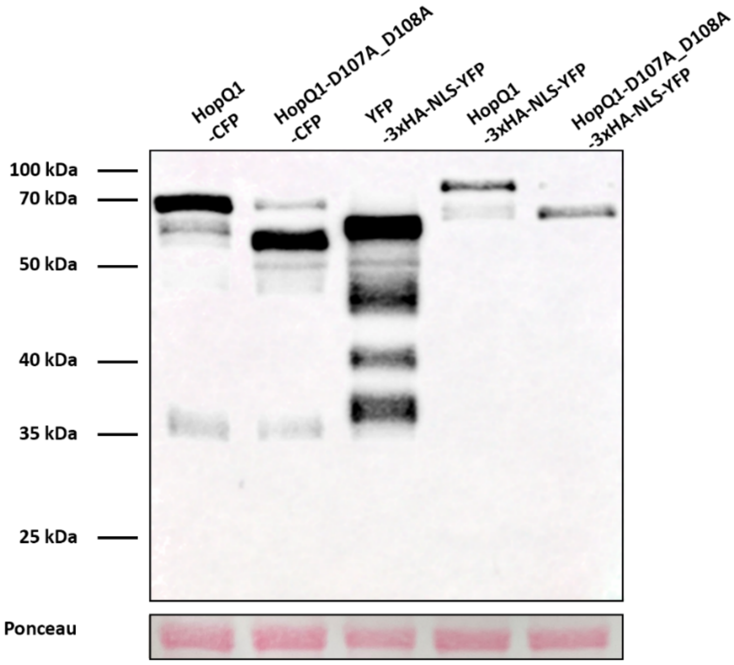

Supplementary Figure S5 The expression level of HopQ1 and YFP variants used in MILo assay. Total protein extracts from agroinfiltrated *N. benthamiana* leaves were subjected to SDS-PAGE, transferred onto a membrane and probed with anti-GFP mouse antibody (dilution 1:1000). Ponceau staining of the membrane was used to demonstrate the equal loading of samples. Source data is provided below.

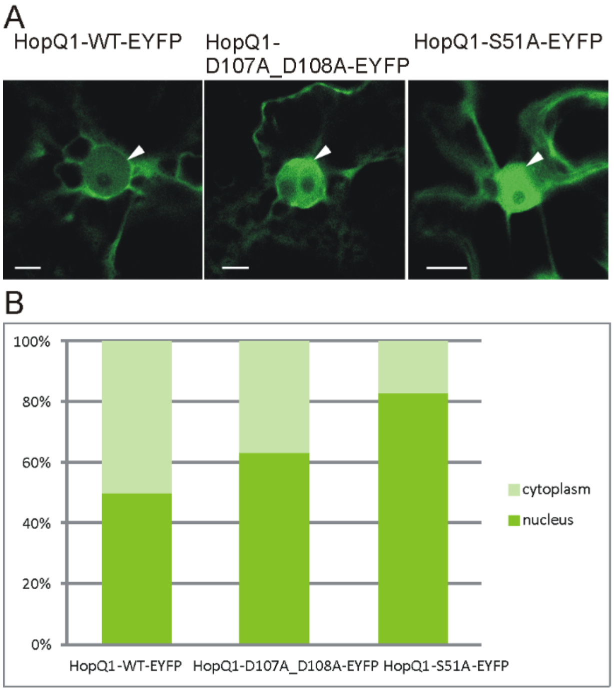

Supplementary Figure S6 Subcellular localization of HopQ1 variants. (A) Confocal images show representative *N. benthamiana* leaf epidermal cells transiently expressing wild-type HopQ1-EYFP or HopQ1-D107A_D108A-EYFP. As a control we added HopQ1-S51A-EYFP , a variant with substitutions in 14-3-3 binding site that is actively translocated to the nucleus (Rymaszewski *et al.*, 2024). White arrowheads indicate the nuclei. The photographs were taken 72 h after agroinfiltration. For each variant, approximately 25 transformed cells were examined. Bars = 10 µm. (B) Relative nuclear and cytoplasmic fluorescence for HopQ1 variants, shown as a ratio of the fluorescence intensity in the given compartment to the total fluorescence intensity in the cell, i.e. intensity in the nucleus plus intensity in the cytoplasm. Average percentage of fluorescence intensities were calculated for yellow fluorescence intensities in the nucleus and cytoplasm determined by ImageJ software. The results were compared statistically using a two-tailed Student’s t test. The differences between all variants are statistically significant at p value < 0.001.

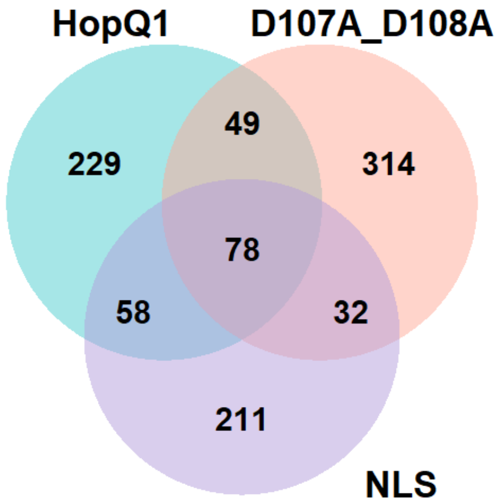

Supplementary Figure S7 The molecular targets of HopQ1 variants. Venn diagram represents common and exclusive subsets of plant proteins purified along with each of the HopQ1 variants and identified by LC-MS/MS analysis. The duplicated and negative control hits were removed. The hits corresponding to each of the effector variant were cross referenced against each other to discern the protein hits unique to the variants tested.

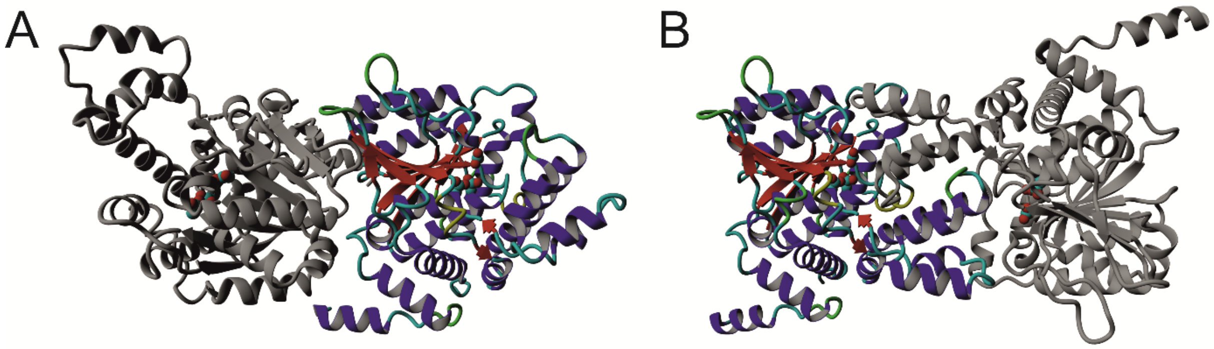

Supplementary Figure S8 Models of HopQ1 (A) and HopQ1-D107A_D108A (B) dimers. Modelling was performed by AlphaFold. Colorful monomers in each dimer are oriented in the same manner in both complexes to show different dimerization interfaces in case of these variants.

Supplementary Table S1. Primers used in this study

Primer name	Sequence	Purpose
**GWB-HA-NLS-YFP**	AGCGCTGGTAGCCCAAAGAAGAAACGGAAGGT CATGGTGAGCAAGGGCGAGGAG	Gateway binary vector containing nuclear localization signal
**HopQ1-C70A-F**	TCAGACGAAAACATGGCCATCAATG	Making of HopQ1-C70A mutant
**HopQ1-C70A-R**	GGTCAGATGAGAGTTTGCGGCTCCCACTTG	
**HopQ1-C230A-F**	GCTCTCATCACTACCGCACCGGATATGGTGC	Making of HopQ1-C230A mutant
**HopQ1-C230A-R**	ATTGATATCACTCATGCCTGCAATTAC	
**q1-mut-NH-F**	TTCACTGATCCTGACAAGGGCCCGGATG	Making of HopQ1-D103,D105A mutant
**RihA-BglII-F**	AGATCTATGCATCGTCCTATCACC	Cloning of RihA encoding sequence into pJET1.2
**RihA-XhoI-R**	CTCGAGAGCGTAAAATTTCAGACGATC	

Source data

Figure S1a

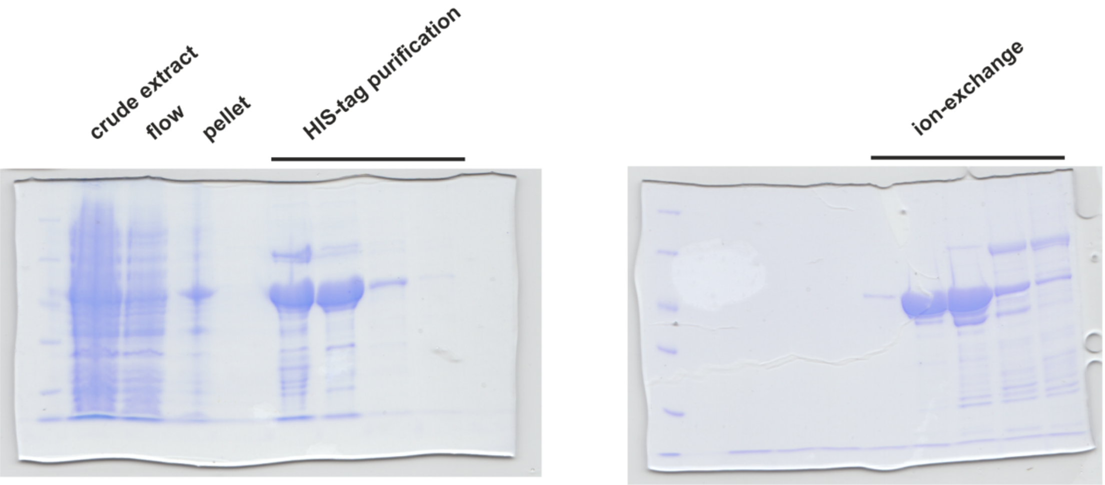

Figure S1b

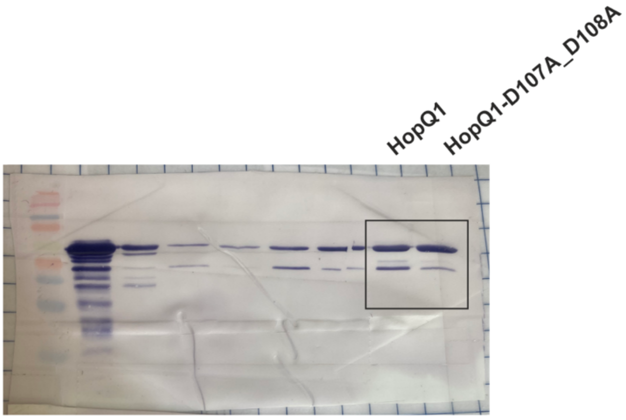

Figure S7

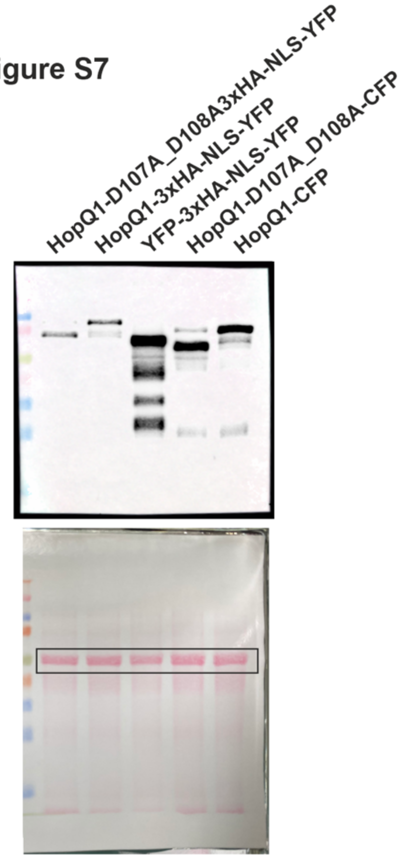

Supplement: Supplementary file 1 — Supplementary Material. [file 41598_2024_82848_MOESM1_ESM.pdf]
